# Impact of *Austropuccinia psidii* (myrtle rust) on Myrtaceae-rich wet sclerophyll forests in south east Queensland

**DOI:** 10.1371/journal.pone.0188058

**Published:** 2017-11-21

**Authors:** Geoff Pegg, Tamara Taylor, Peter Entwistle, Gordon Guymer, Fiona Giblin, Angus Carnegie

**Affiliations:** 1 Horticulture & Forestry Science, Department of Agriculture & Fisheries, Brisbane, Queensland, Australia; 2 Plant Biosecurity Cooperative Research Centre, Bruce, ACT, Australia; 3 Griffith University, Nathan, Queensland, Australia; 4 North East Agriculture Services, New South Wales, Australia; 5 Queensland Herbarium, Department of Science, Information Technology and Innovation, Mt Coot-tha, Queensland, Australia; 6 NSW Department of Primary Industries, NSW Forest Science, Parramatta, New South Wales, Australia; Universita degli Studi di Pisa, ITALY

## Abstract

In April 2010, *Austropuccinia psidii* (formerly *Puccinia psidii*) was detected for the first time in Australia on the central coast of New South Wales. The fungus spread rapidly along the east coast and can now be found infecting vegetation in a range of native forest ecosystems with disease impacts ranging from minor leaf spots to severe shoot and stem blight and tree dieback. Localised extinction of some plant species has been recorded. In 2014, the impact of *A*. *psidii* was observed for the first time in a wet sclerophyll site with a rainforest understory, dominated by species of Myrtaceae, in Tallebudgera Valley, south east Queensland, Australia. This study aimed to determine the impact of *A*. *psidii* on individual species and species composition. Here we provide quantitative and qualitative evidence on the significant impact *A*. *psidii* has in native ecosystems, on a broader range of species than previously reported. *Archirhodomyrtus beckleri*, *Decaspermum humile*, *Gossia hillii* and *Rhodamnia maideniana* are in serious decline, with significant increases in tree mortality over the period of our study. This research further highlights the potential of this invasive pathogen to negatively impact native ecosystems and biodiversity.

## Introduction

*Austropuccinia psidii* (formerly *Puccinia psidii*) [1] (myrtle rust) is a serious pathogen whose rapid global spread impacts commercially and ecologically important species of Myrtaceae [2]. It was first reported from Brazil in 1884 infecting *Psidium guajava* [3] and now occurs in numerous South and Central American countries [2], as well as USA (Hawaii, Florida, California) [4,5,6], Australia [7], New Caledonia [8,9], South Africa [10], Indonesia [11] Singapore [12] and most recently New Zealand (www.mpi.govt.nz: Accessed 5 September 2017). The implications of this global spread are still largely undetermined.

*Austropuccinia psidii* was detected in Australia in April 2010 on the central coast of New South Wales (NSW) [7]. Many naïve species of Myrtaceae in Australia are susceptible hosts such as eucalypt (*Eucalyptus*, *Corymbia*), paperbark and bottlebrush (*Melaleuca*), tea tree (*Leptospermum*) and lilly pilly (*Acmena*, *Syzygium*). Myrtaceae are the dominant iconic and ecologically important plant species in Australia [13], with approximately 2,250 native species within 88 genera, representing more than half of the global number of Myrtaceae [14]. There are more species of Myrtaceae than any other plant family in Australia [15]. Species of Myrtaceae are present in 11 out of the 13 major vegetation formations in Australia [16]. A range of vertebrates and invertebrates take nectar and pollen from inflorescences and fruits, and fleshy-fruited species provide a food source for a range of birds and mammals [13].

Since its initial detection in NSW, *A*. *psidii* spread rapidly along the east coast of Australia, from southern NSW, as far north as Bamaga at the tip of Cape York Peninsula, Queensland [17,18]. It is not reported to have established in native ecosystems in Victoria despite being detected in nurseries and gardens in 2011 (D. Smith Pers. Comm.). More recently (January 2015) *A*. *psidii* was detected in Tasmania (http://dpipwe.tas.gov.au) and the Northern Territory [19].

*Austropuccinia psidii* is now identified from a range of native forest ecosystems in Australia including coastal heath, coastal and river wetlands, sand island ecosystems, and littoral, montane, subtropical and tropical rainforests [17]. Its host range exceeds 350 species from 58 genera in Australia alone [2]. Symptoms of infection by *A*. *psidii* range from minor leaf spots to severe foliage and stem blight, as well as infection of flowers and fruit of some species. Severe infection and crown loss, dieback and tree mortality has been reported for two species of widespread distribution in NSW and Queensland—*Rhodamnia rubescens* and *Rhodomyrtus psidioides*—across their entire native range [18]. While both species are now in decline, impact on *R*. *psidioides* is particularly severe with deaths of over half the trees in many stands, including mature trees up to 12 m tall. This level of decline occurred within two to three years following establishment of *A*. *psidii* and both species have now been listed as Critically Endangered in NSW due to *A*. *psidii* (NSW Scientific Committee 2017—http://www.environment.nsw.gov.au/committee/preliminarydeterminationsbydate.htm Accessed 5 September 2017). The perceived threat of *A*. *psidii* to Australian biodiversity and industry is now being realised. However, there is a paucity of information on the impact of *A*. *psidii* on other native Myrtaceae in Australia.

This study was designed to examine levels of impact on a broader range of species of Myrtaceae, and to determine whether these impacts could alter the plant composition and community structure within native ecosystems. An area of subtropical wet sclerophyll forest with rainforest understory dominated by Myrtaceae was identified on private property (Ryans Road) in the Tallebudgera Valley, south east Queensland, Australia (28°12´7.28´S, 153°21´0.66E, Altitude 80 m asl). The site has a history of forest logging activities and clearing for cattle grazing, but has since been allowed to regenerate naturally. This site was initially assessed for impact of *A*. *psidii* on *Rhodomyrtus psidioides* and *Rhodamnia rubescens* in 2014 [18] and was revisited in 2016 to examine the progression of decline on these species. At this time, significant levels of infection and related decline were identified on a range of other Myrtaceae.

## Materials and methods

### Ryans Road study site

To determine impact of *A*. *psidii* on the range of different Myrtaceae species within the site, four 50 m long x 2m wide line transects were assessed. Location of transects was non-random, and based on site aspect and selected to ensure data on a range of Myrtaceae species within the site was captured. The start and finish of each transect was marked and GPS co-ordinates recorded. Trees one metre each side of the centre line were marked with flagging tape and individually numbered. Only species of Myrtaceae were marked, but the number of non-Myrtaceae within each transect was recorded and identifications conducted for the most common species. Samples and photos of Myrtaceae and non-Myrtaceae were taken to confirm species identification.

Each tree was recorded according to position within the canopy as:

- Over-storey—trees >15m in height. Trees at this height were assessed with the aid of binoculars
- Mid-storey—trees ≥ 4 m but < 15 m in height
- Under-storey—trees < 4 m but > 1 m in height
- Regeneration—seedlings and saplings ≤ 1 m in height

*Austropuccinia psidii* disease impact on individual trees was assessed as:

- Percentage of dead branches
- Percentage of the remaining branches with evidence of dieback (including defoliation). To compare levels of branch dieback within and between species, trees where all branches were rated as dead were removed prior to analysis.
- Crown transparency score as an indicator of overall tree health and to enable monitoring of change over time for mid and over-story species only. Transparency was assessed as per Carnegie *et al*. [18] using the foliage scale developed by Schomaker *et al*. [20].

Disease incidence and severity levels were also assessed as described previously [18,21], but due to inconsistent levels of growth flush between species the data was not included in this study. The number of dead Myrtaceae within our study transects was also recorded and then recounted again 12 months later (August 2017) to determine rates of decline for individual species.

### Comparison site assessments

**Fig 1:**
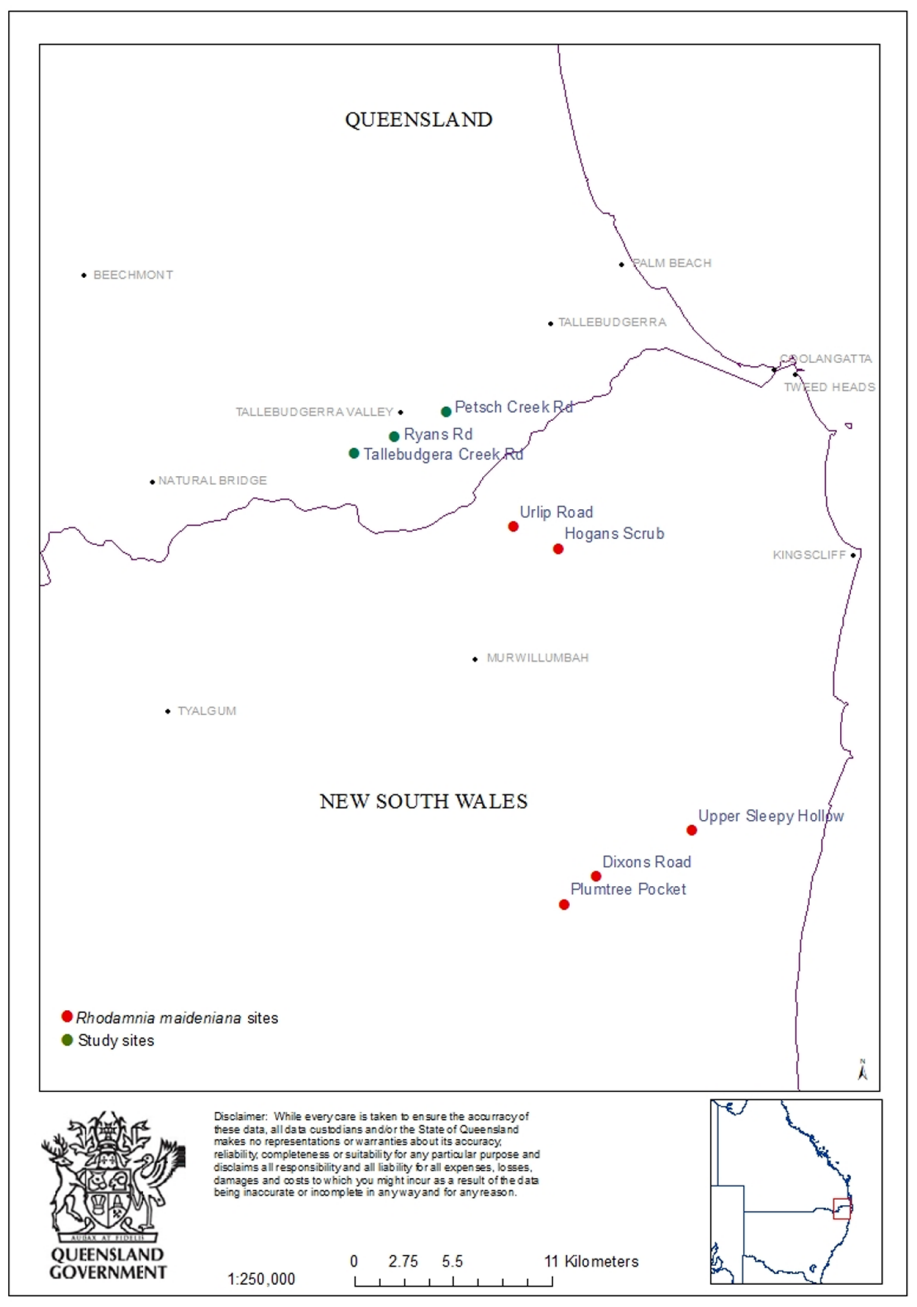
Map showing the location of study sites in Tallebudgera Valley and northern New South Wales.

To determine if the level of disease and associated dieback was unique to the Ryans Road site or more widespread, other plant communities in the area were examined for presence of myrtle rust related dieback. Two sites (Tallebudgera Creek Road, 28°12´37.96S; 153°19´50.32E; 96 m asl, two kilometres north-west from Ryans Road site; Petsch Creek Road, 28°11´21.04S; 153°22´35.66E; 77 m asl, three kilometres south-east of Ryans Road site) with similar over-story species to Ryans Road were assessed along two randomly selected transects (50 m long x 2m wide) with trees rated for disease and decline levels as described previously (Fig 1).

### Observations of *Austropuccinia psidii* impact on other Myrtaceae

A range of other Myrtaceae species, in addition to those occurring within the transect plots, were identified and assessed for *A*. *psidii* impact at the Ryans Road site. Species assessed included *Syzygium hodgkinsoniae* (listed as a Vulnerable—http://www.ala.org.au), and *Syzygium corynanthum*, which is considered common in the region.

### Decline over time

*Rhodomyrtus psidioides*, *Rhodamnia rubescens* and *R*. *maideniana* were assessed for *A*. *psidii* impact at the Ryans Road site in 2014 [18], although data for *R*. *maideniana* was not previously published. In 2016, as part of this current study, the same trees were re-assessed to determine rates of decline. Impact data for *Rhodamnia maideniana* that was also collected from five sites across northern NSW (Fig 1) but not previously published, also is reported. These sites are located within 30km to the south of Tallebudgera Valley. At the Ryans Road site, photographic evidence of decline was also captured enabling comparison of trees from 2014 to 2016. Photos of other Myrtaceae (*Acmena smithii*, *Decaspermum humile*, *Syzygium corynanthum*) taken in 2014 were re-taken in 2016 as a visual comparison of decline caused by *A*. *psidii*.

### Analysis

Data from the four transects at Ryans Road were pooled for analysis, with individual trees of each species considered as replicates for between-species comparisons. Normality of data and equality of variance were assessed using an F test. All proportion data were Arcsine square root transformed prior to analysis using one-way ANOVA and compared using Fishers PLD post hoc test (Statview^®^). Back-converted data were used to present data.

### Study site access

Where studies were conducted on private property permission to access the site and conduct the research was granted by the landowner. Other sites accessed including public reserves and state owned conservation areas were covered within current permits. All studies conducted were non-destructive surveys and assessments. Samples of plant material for identification were done as per Queensland Herbarium permit requirements.

## Results

### Plant species composition and *Austropuccinia psidii* impact—Ryans Road

#### Non-Myrtaceae v Myrtaceae

**Fig 2:**
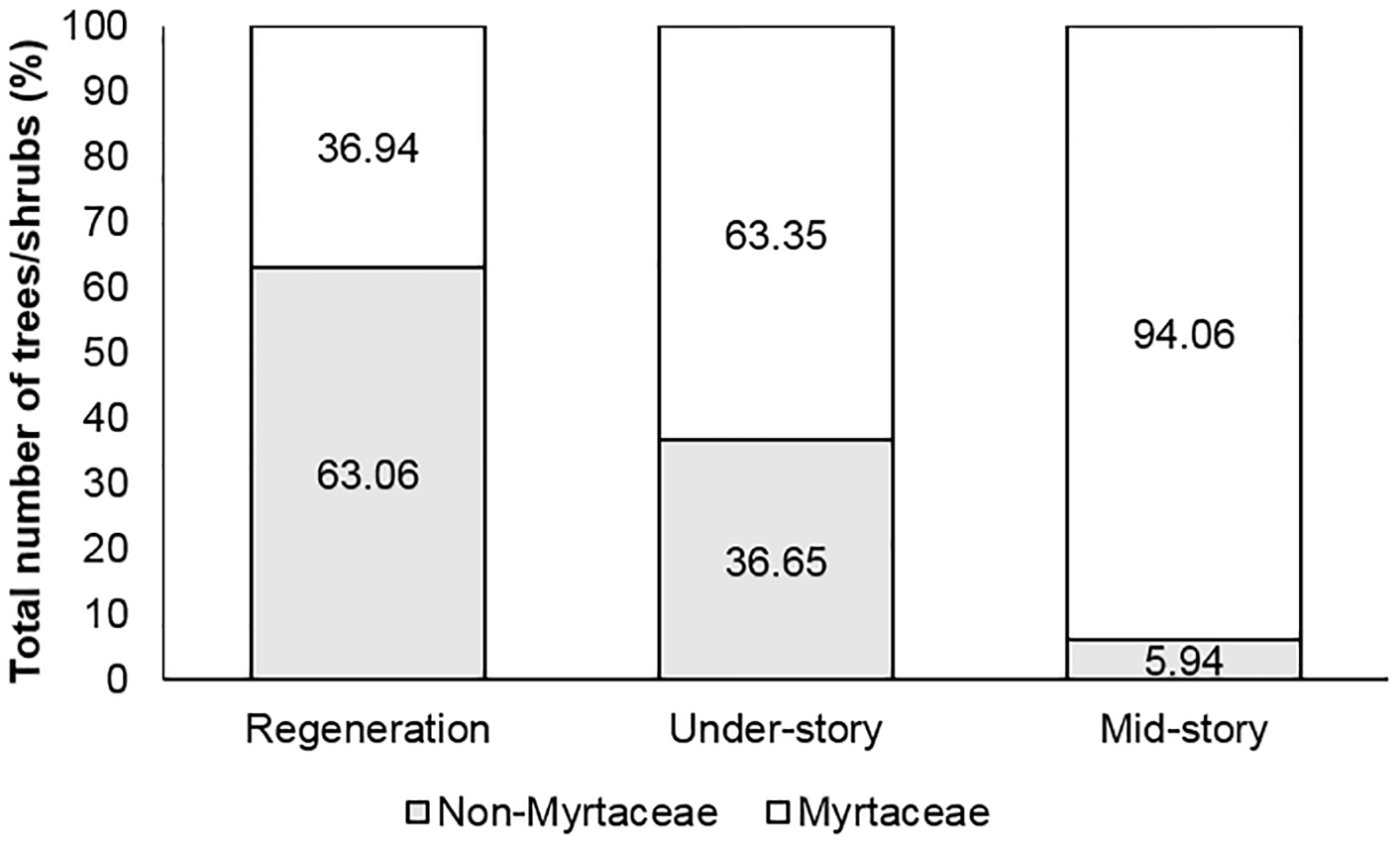
Composition of Myrtaceae and non-Myrtaceae (n = total number of trees/shrubs) within the different forest layers in a subtropical rainforest/wet sclerophyll ecosystems at Site 1, Ryans Road, Tallebudgera Valley, Queensland.

The vegetation in the mid- and under-story was dominated by Myrtaceae (75%) with an over-story consisting primarily of *Eucalyptus grandis* and *Lophostemon suaveolans* (Fig 2). However, non-Myrtaceae dominated the make-up of the regenerating seedlings and saplings.

The majority of non-Myrtaceae species within the plots were present as regenerating seedlings (72%), followed by species present in the under-story (25%). However, 78% of the under-story was established tree ferns (*Cyathea leichhardtiana*, Cyatheaceae), which are suited to growing in heavily shaded environments. The other most common seedling and sapling species present in all plots was *Neolitsea dealbata* (Lauraceae), a common species in wet sclerophyll and rain forest ecosystems, favoured by disturbance. Weed species identified as emerging seedlings included *Ochna serrulata* (Ochna) (Ochnaceae) and *Cinnamomum camphora* (Camphor laurel) (Lauraceae).

*Decaspermum humile*, *Archirhodomyrtus beckleri* and *Gossia hillii* were species of Myrtaceae that dominated the mid- and under-story. *Rhodamnia maideniana* was common in the under-story. *Acmena smithii* was also locally common, particularly in the open forest edges, as was *Rhodamnia rubescens* and *Rhodomyrtus psidioides*. Large rainforest trees present in the open areas and more established forest areas include *Syzygium hodgkinsoniae* and *S*. *corynanthum*, with some trees exceeding 20 metres in height. Many of the Myrtaceae species at this site have previously been described as susceptible to *A*. *psidii* (Table 1).

**Table 1:** Myrtaceae species at Ryans Road, Tallebudgera Valley and previous *Austropuccinia psidii* susceptibility ratings [17].

Host species	Canopy Position	Rust susceptibility
*Eucalyptus grandis*	Over-story	Relatively Tolerant-Moderate Susceptibility
*Lophostemon confertus*	Over-story	Resistant
*Syzygium oleosum*	Mid-story/Forest edge	Highly Susceptible
*Syzygium corynanthum*	Over-story/Forest edge	Relatively Tolerant
*Syzygium hodgkinsoniae*	Mid-story/Forest edge	Not Rated prior to 2014
*Rhodomyrtus psidioides*	Forest edge	Extremely Susceptible
*Rhodamnia rubescens*	Forest edge	Highly—Extremely Susceptible
*Rhodamnia maideniana*	Understory/Forest edge	Extremely Susceptible
*Decaspermum humile*	Mid-story/Forest edge	Extremely Susceptible
*Archirhodomyrtus beckleri*	Mid-story	Not Rated prior to 2014
*Gossia hillii*	Mid-story	Highly—Extremely Susceptible
*Acmena smithii*	Mid/Under-story/forest edge	Relatively Tolerant—Moderate Susceptibility
*Pilidiostigma glabrum*	Under-story	Relatively Tolerant—Moderate Susceptibility

#### Regeneration

**Fig 3:**
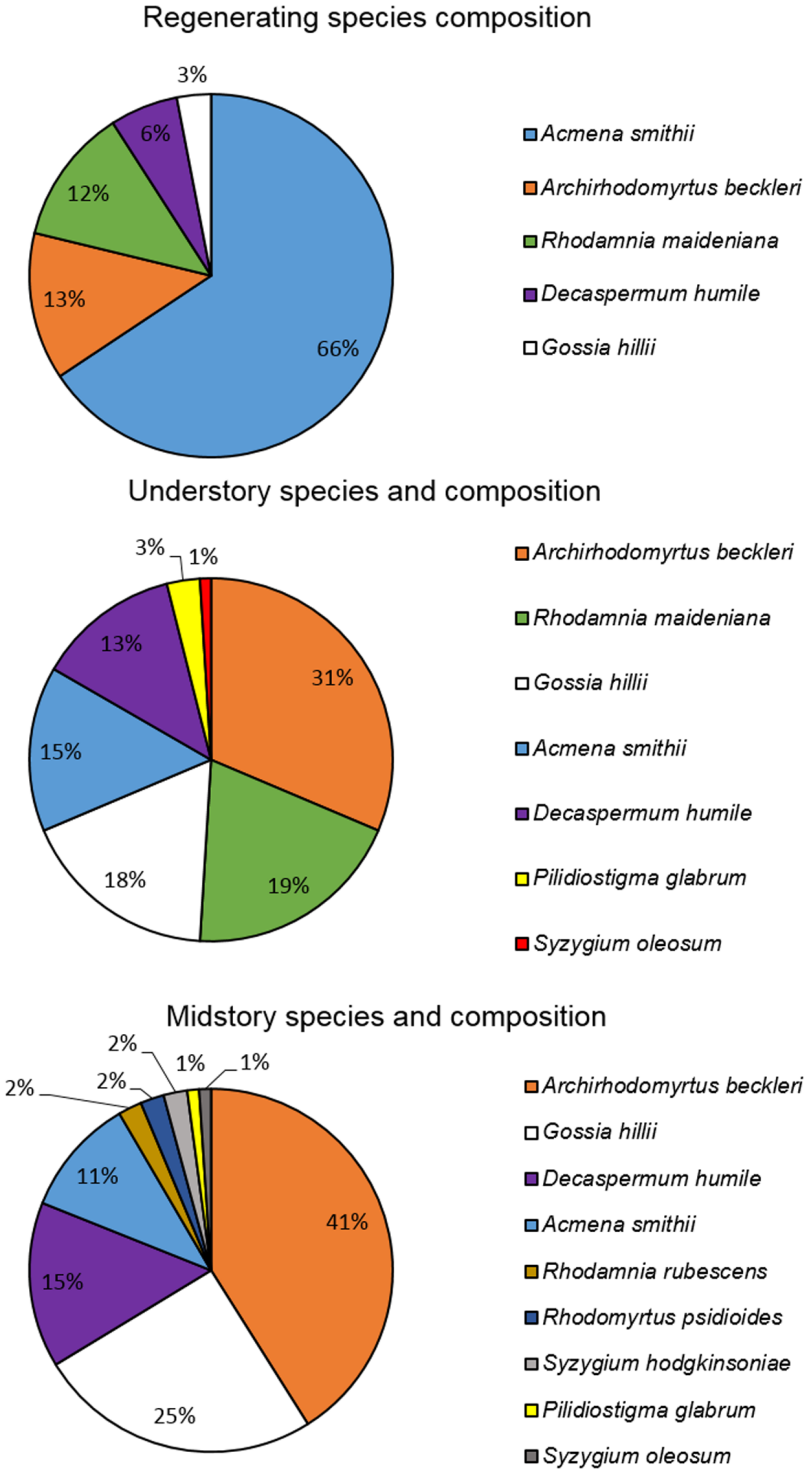
Composition of regenerating (n = 99), under-storey (n = 101) and mid-story (n = 93) Myrtaceae in 2016 within a subtropical rainforest/wet sclerophyll forest at Ryans Road, Tallebudgera Valley, Queensland.

*Acmena smithii* (n = 65) was the most common species regenerating at Ryans Road, making up 66% of all the Myrtaceae seedlings and small saplings assessed (Fig 3) and showing significantly lower levels of *A*. *psidii* related branch death (F_4,94_ = 5.637; P = 0.0004) and dieback (F_4,94_ = 120.44; P<0.0001) (Table 2). Of the *A*. *smithii* assessed, 72% were regenerating seedlings or saplings with significantly lower branch death (F_2,87_ = 6.15; P = 0.03) levels in comparison to more mature trees in the under- (P = 0.02) and mid-story (P = 0.004).

**Table 2:** Impact of *Austropuccinia psidii* infection on the main species making up the three vegetation-story components of the wet sclerophyll/rainforest ecosystem at Ryans Road, Tallebudgera Valley, Queensland. The composition within the forest canopy is presented for each species including Regeneration, Under-story and Mid-story. Impact levels are based on the mean percentage of dead branches, the mean percentage of remaining branches with evidence of dieback as a result of infection by *A*. *psidii*. Mean crown transparency is used as an indicator of tree health. The same letters within columns indicate that means do not differ significantly when comparing species (Capital letters = Regeneration; Capital Bold = Under-story; Lower case = Mid-story) (S1 Table). The same numbers within columns indicate that means do not differ significantly when comparing disease impact levels within species at the different canopy positions (S1 Table).

Tree species	Canopy position	Composition within canopy (% per species)	Branch death (%)	Branch dieback (%)	Transparency
*Acmena smithii*	Regeneration	72.22	0.08 ^±0.08^ A,1	3.23 ^±1.44^ A,1	-
	Under-story	16.67	6.67 ^±5.16^ **A**,2	13.33 ^±6.7^ **A**,12	50.91 ^±10.48^ **A**,1
	Mid-story	11.11	8 ^±5.28^ a,2	21 ^±13.2^ a,2	65.5 ^±5.4^ a,1
*Archirhodomyrtus beckleri*	Regeneration	15.48	8.85 ^±4.53^ B,1	93.85 ^±2.84^ B,1	-
	Under-story	38.1	43.75 ^±6.61^ **B**,2	97.66 ^±2.34^ **B**,1	93.45 ^±1.57^ **BC**,1
	Mid-story	46.42	22.56 ^±5.42^ a,1	91.43 ^±3.39^ b,1	89.49 ^±1.42^ b,2
*Decaspermum humile*	Regeneration	18.18	0 ^±0^ A,1	33.33 ^±21.08^ C,1	-
	Under-story	39.4	48.46 ^±11.03^ **B**,2	100 ^±0^ **B**,2	95.61 ^±1.21^ **B**,1
	Mid-story	42.42	86.79 ^±6.13^ b,3	100 ^±0^ b,2	98.21 ^±0.66^ c,1
*Gossia hillii*	Regeneration	6.82	0 ^±0^ A,1	100 ^±0^ B,1	-
	Under-story	40.91	33.89 ^±8.22^ **B**,1	96.87 ^±3.12^ **B**,1	91.11 ^±2.04^ **BC**,1
	Mid-story	52.27	42.61 ^±7.99^ c,1	100 ^±0^ b,1	94.78 ^±1.28^ c,1
*Rhodamnia maideniana*	Regeneration	61.29	2.92 ^±2.5^ A,1	100 ^±0^ C,1	-
	Under-story	38.71	4.74 ^±1.69^ **A**,1	93.42 ^±5.35^ **B**,1	85.28 ^±2.08^ **C**
	Mid-story	0	-	-	-

Regenerating seedlings and saplings were less common for *A*. *beckleri* (15%) (n = 13), *D*. *humile* (n = 6) (18%) and *G*. *hillii* (n = 3) (7%) (Table 2). Branch death was highest on *A*. *beckleri* seedlings and saplings. *Archirhodomyrtus beckleri*, *R*. *maideniana* (n = 12) and *G*. *hillii* all had significant levels of *A*. *psidii* related dieback recorded. *Decaspermum humile* made up 6% of the regenerating Myrtaceae, and although some of those seedlings had only low levels of decline with no branch death, an average one-third of branches had dieback symptoms.

#### Under-story

Species composition in the under-story of the wet sclerophyll/rainforest plots at Ryans Road were made up of seven different Myrtaceae (Fig 3). The under-story was, dominated by *A*. *beckleri* (n = 32) and *Rhodamnia maideniana* (n = 19), *G*. *hillii* (n = 18), *A*. *smithii* (n = 15) and *D*. *humile* (n = 13) were also relatively common. There were only three *Pilidiostigma glabrum* (n = 3) and one *S*. *oleosum* (n = 1) that occupied this vegetation layer within the study transects. Due to the low numbers of individuals present, assessment of both *P*. *glabrum* and *S*. *oleosum* were removed from the data prior to analysis.

Significant differences in disease impact were identified when comparing levels of branch death between species (F_4,92_ = 9.356; P<0.0001) (Table 3), with the lowest levels recorded for *R*. *maideniana* and *A*. *smithii*. *Decaspermum humile* (P<0.0001), *A*. *beckleri* (P<0.0001) and *G*. *hillii* (P = 0.006) had significantly higher levels of branch death than *R*. *maideniana* and *A*. *smithii*. Branch death levels for *D*. *humile*, *A*. *beckleri* and *G*. *hillii* were not significantly different from each other.

**Table 3:** Impact of *Austropuccinia psidii* based on a single assessment conducted in 2014of *Rhodamnia maideniana* trees across six sites in NSW and Queensland. The same letters within columns indicate that means do not differ significantly.

Location	Infected new growth (%)	Transparency
Hogans Scrub, NSW	63.75^±11.33^a	42.5^±4.53^a
Urlip Road, NSW	91.54^±4.61^b	48.85^±3.5^a
Upper Sleepy Hollow, NSW	100^±0^b	52.33^±3.16^a
Plumtree Pocket, NSW	97.78^±1.21^b	52.78^±5.47^a
Dixons Road, NSW	97.65^±0.87^b	53.82^±4.61^a
Tallebudgera Valley, Qld	100^±0^b	68.75^±3.55^b

Branch dieback assessments were based on the percentage of living branches showing evidence of shoot death and foliage loss and were often typified by the presence of epicormic shoots occurring along woody branches. The degree of severity of dieback was not classified in this study. Significant differences in levels of branch dieback were identified (F_4,79_ = 57.409; P<0.0001). *Acmena smithii* had significantly lower levels of branch dieback than all other species identified in the forest under-story (P<0.0001) (Table 3). Despite having low levels of branch death recorded, dieback levels of *R*. *maideniana* averaged 93%, slightly lower than *D*. *humile*, *G*. *hilli* and *A*. *beckleri* but not significantly different (Table 3). The effects of repeated rust infection on *R*. *maideniana* were typified by epicormic regeneration along the woody branches and “witches broom–like” appearance at the branch tips.

Of the three *P*. *glabrum* trees assessed, all had some level of branch death and rust related branch dieback. No branch death was observed on the single *S*. *oleosum* tree, although infection was identified on foliage and evidence of shoot dieback recorded.

#### Mid-story

**Fig 4:**
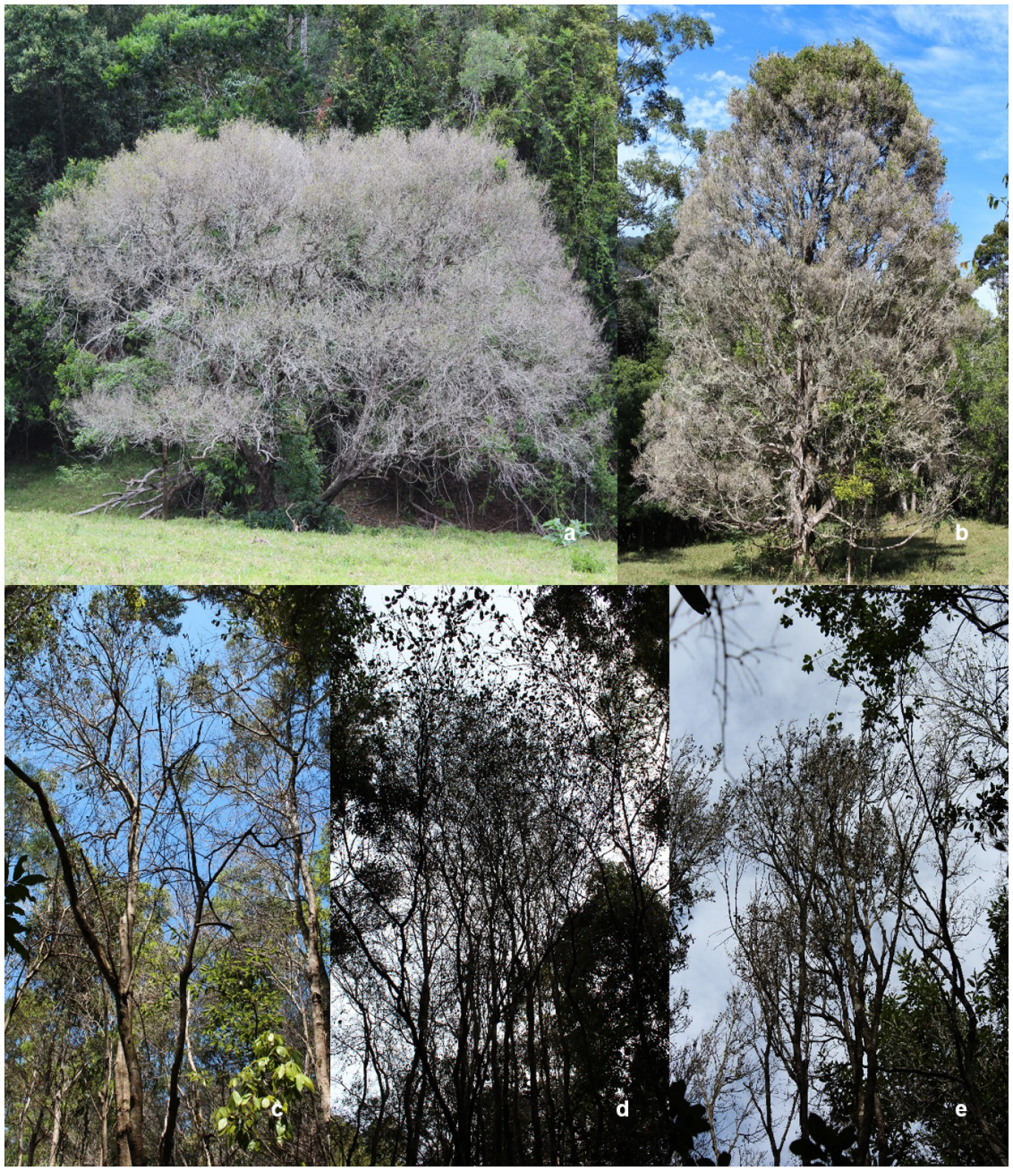
Tree dieback and increased canopy transparency as a result of repeated infection by *Austropuccinia psidii* on species of Myrtaceae (a) *Decaspermum humile*; (b) *Syzygium corynanthum*; (c) *Archirhodomyrtus beckleri*; (d) *Syzygium hodgkinsoniae*; (e) *Gossia hillii*.

*Archirhodomyrtus beckleri* (n = 39) was the most common species in the mid-story making up 41% of all the Myrtaceae identified (Fig 3). *Gossia hillii* (n = 23), *D*. *humile* (n = 14) and *A*. *smithii* (n = 10) were the next most common species. Due to their low numbers within plots, *R*. *rubescens* (n = 2), *R*. *psidioides* (n = 2), *S*. *hodgkinsoniae* (n = 2), *P*. *glabrum* (n = 1) and *S*. *oleosum* (n = 1) were excluded from the analysis. All *R*. *rubescens* and *R*. *psidioides* trees were dead, while *S*. *hodgkinsoniae* had evidence of dieback on all branches with significant foliage loss and infection occurring on all new growth flush (Fig 4). Epicormic shoot regrowth was common on *S*. *hodgkinsoniae*, which was also infected by *A*. *psidii*.

Significant differences in levels of branch death and branch dieback were recorded for the main Myrtaceae present in the mid-story (Fig 4). Using crown transparency as an indicator of health, significant differences were identified (F_3,82_ = 23.213; P<0.0001) (Table 2). *Acmena smithii* had the lowest levels of crown transparency indicating healthier trees, significantly lower than *A*. *beckleri*, *D*. *humile* and *G*. *hillii* (P<0.001). *Decaspermum humile* had the highest transparency levels, indicating poor tree health, significantly higher than *A*. *beckleri* (P = 0.0001) but not *G*. *hillii* (P = 0.0794).

Branch death (F_3,82_ = 16.226; P<0.0001) and dieback (F_3,64_ = 34.37; P<0.0001) levels also differed significantly between species. Branch death levels were significantly higher on *D*. *humile* in comparison to *A*. *smithii* (P<0.0001), *A*. *beckleri* (P<0.0001) and *G*. *hillii* (P = 0.0004). While the level of branch death recorded for *A*. *beckleri* in the mid-story trees was relatively low and not significantly different to *A*. *smithii* (P = 0.1666), dieback was recorded on >90% of the remaining living branches, significantly higher than what was recorded for *A*. *smithii* (P<0.0001). Similar dieback levels were recorded for *D*. *humile* and *G*. *hillii*.

**Fig 5:**
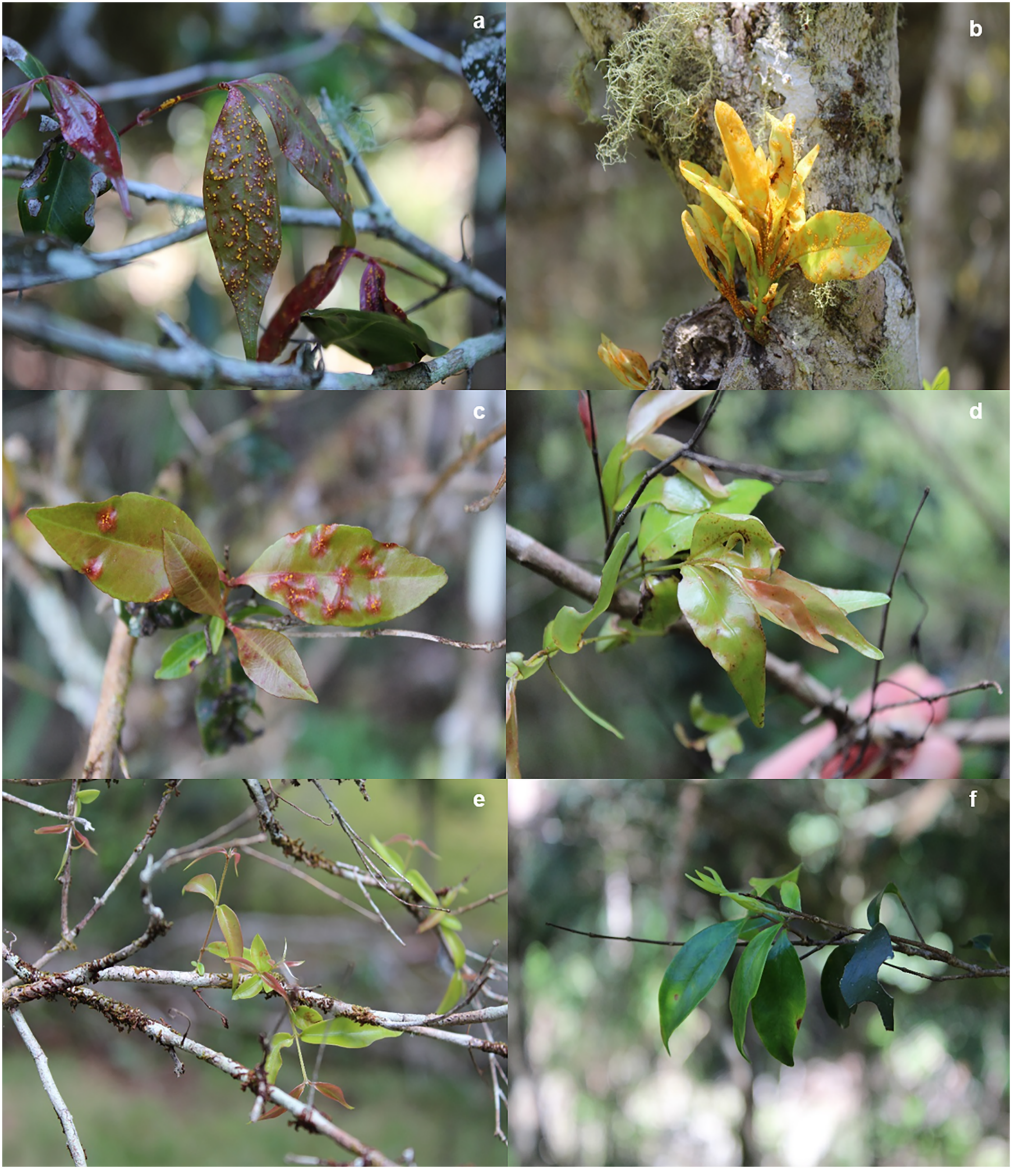
Symptoms of *Austropuccinia psidii* infection of epicormic/reshooting young foliage of (a) *Rhodamnia maideniana*, (b) *Syzygium corynanthum*, (c*) Syzygium hodgkinsoniae*, (d) *Gossia hillii*, (e) *Decaspermum humile* and (f) *Archirhodomyrtus beckleri*.

Epicormic shoot regeneration, an indicator of stress, was found on the main trunk and base of *G*. *hillii*, *D*. *humile* and *A*. *beckleri* trees showing A. *psidii* related dieback. In many cases, the epicormic shoots were also infected by *A*. *psidii* and dying back (Fig 5).

No symptoms of *A*. *psidii* infection or related dieback was observed on the over-story species *E*. *grandis* or *L*. *confertus*.

#### Tree deaths

Assessments of species decline from 2016 to 2017 show a dramatic increase in tree mortality levels. Tree deaths from 2016 to 2017 (12 months) increased three-fold for *Archirhodomyrtus beckleri* (13% to 44%) and more than doubled for *Decaspermum humile* (36% to 73%) and *Gossia hillii* (18% to 38%). No deaths were recorded for either *Acmena smithii* or *Rhodamnia maideniana* within the study transects, despite significant levels of dieback recorded on *R*. *maideniana*.

### Impact of *Austropuccinia psidii*–Tallebudgera Creek Road, Petsch Creek Road

Two additional sites within the Tallebudgera Valley, Tallebudgera Creek Road and Petsch Creek Road, were examined to determine if the impact levels of *A*. *psidii* identified at our primary study site (Ryans Road) were representative of impact on a larger scale in subtropical rainforest/wet sclerophyll environments. At the Tallebudgera Creek Road site over-story species were primarily *E*. *grandis* and *L*. *confertus* with *A*. *beckleri* the dominant mid- and under-story species and *S*. *oleosum* scattered throughout the site. *Tristaniopsis laurina* was found on the margins of the forest but were not assessed. At the Petsch Creek Road site, *A*. *beckleri* also dominated the under- and mid-story with *R*. *rubescens* and *R*. *psidioides* identified on the margins. *Eucalyptus grandis* was the dominant over-story species. At both sites, non-Myrtaceae species were absent from the mid- and under-story but were represented as seedlings (regeneration).

At both sites, considerable levels of decline in the under- and mid-story were identified with particularly severe impact on *A*. *beckleri*. Of the 87 *A*. *beckleri* trees assessed at the Tallebudgera Creek Road site, 79% of trees were dead. The remaining trees showed evidence of branch death and dieback on living branches. No *A*. *beckleri* seedlings were located at the time of assessment. *Syzygium oleosum* was scattered within the site, with four trees having some level of *A*. *psidii* infection on new growth flush. A low percentage (<25%) of branch dieback was identified on all *S*. *oleosum* trees.

At the Petsch Road site, the level of decline in *A*. *beckleri* was less severe. Of the 23 *A*. *beckleri* trees assessed, all had some branch dieback but no evidence of branch death. No seedlings of *A*. *beckleri* were located across the site. Nine dead *R*. *psidioides* trees were also identified, with no evidence of coppice or seedling regeneration observed. A single *R*. *rubescens* tree was identified on the forest edge with 25% of branches dead and the remaining branches having evidence of dieback and foliage loss.

### Observations of *Austropuccinia psidii* impact on other Myrtaceae

#### Syzygium hodgkinsoniae

Saplings and mature *S*. *hodgkinsoniae* trees were assessed outside the established study plots. All juvenile (saplings) trees had very high incidence (90–100%) of rust infection on new shoots and expanding foliage. Dieback on all branches is likely to have been caused by past infection episodes (Figs 4 and 5) with symptoms typical of *A*. *psidii* infection evident.

The impact of *A*. *psidii* on mature *S*. *hodgkinsoniae* trees was less obvious as foliage/crown density levels were high with trees examined having a low transparency recorded. However, the presence of branch dieback, dead growing tips on most branches and epicormic shoots was evidence of stress. These epicormic shoots were infected by *A*. *psidii*. Despite the level of dieback, fruit was present on one of the trees. No evidence of *A*. *psidii* infection was identified on the fruit at the time.

#### Syzygium corynanthum

Twenty *S*. *corynanthum* trees were identified at the Ryans Road study site but outside of our study plots. Three large (25m+ in height) *S*. *corynanthum* trees were present in an open area adjacent to the study plots. One tree showed significant levels of decline with 75% defoliation, 20% branch death and the remaining branches showing evidence of dieback (Fig 4). Coppice shoots on branches had evidence of older infection causing dieback and fresh infection on new shoots (Fig 5). On the other two trees examined, foliage loss was restricted to dieback at the very tip of branches and foliage loss was less obvious. However, high levels of *A*. *psidii* infection were observed on new growth on both trees despite the overall low levels of decline in tree health. The remaining individuals were over-story trees on the forest edge along the ridge that leads to a creek where they are more established and evidence of historical site disturbance is less obvious. The trees on the forest edge all had some evidence of infection occurring on branch tips. Dieback ranged from 10–50% of branches on most trees, and only two trees with low levels (>10%) of branch death.

### Decline of species over time

**Fig 6:**
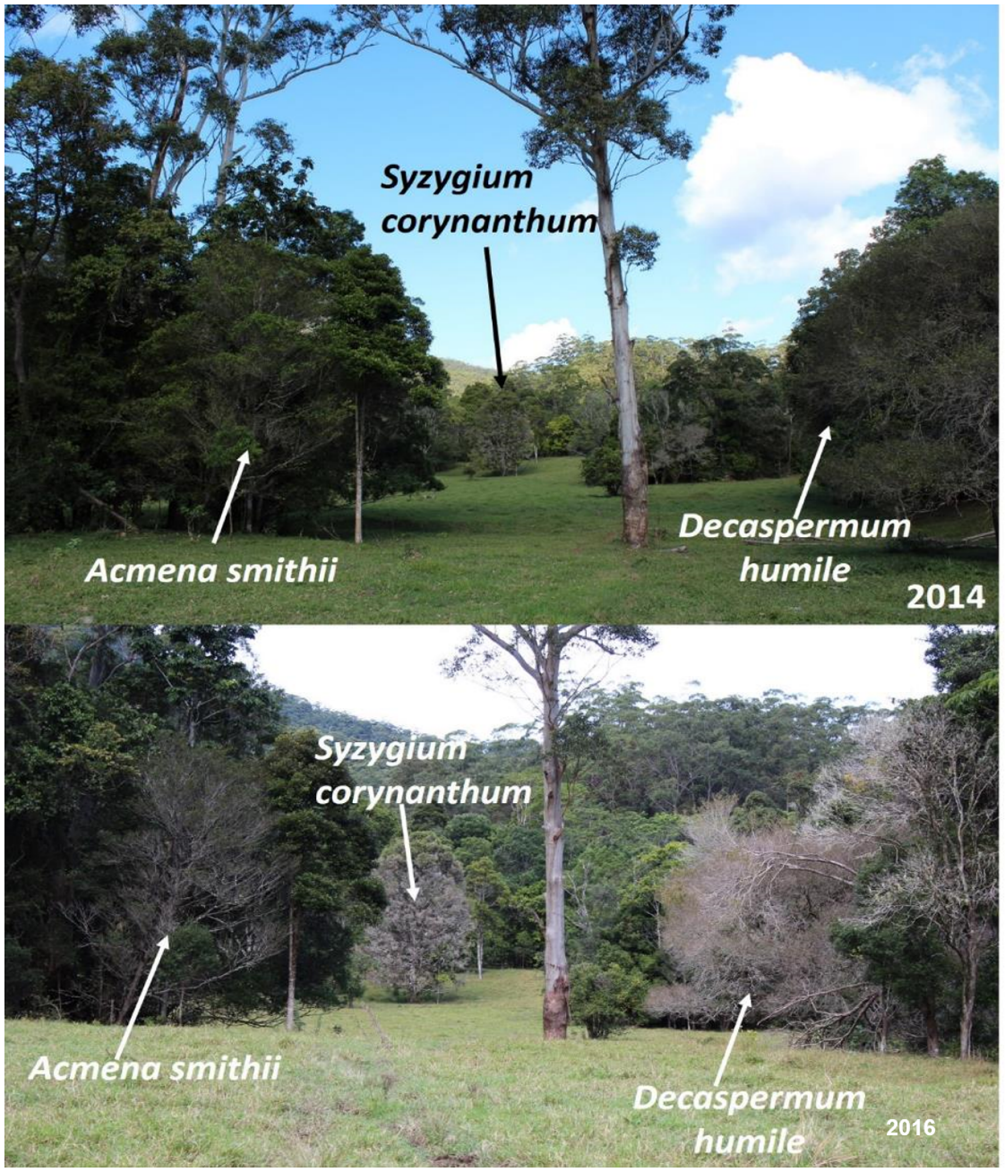
Progression of decline from 2014 (top) to 2016 (bottom) on *Acmena smithii*, *Decaspermum humile* and *Syzygium corynanthum* caused by repeated *Austropuccinia psidii* infection.

A comparison of photographs taken in 2014 and 2016 indicate a decline in the health of the Myrtaceae at Ryans Road site. Photographs taken in 2014 (Fig 6) show some evidence of disease impact on *D*. *humile* and *S*. *corynanthum* but little evidence of impact on *A*. *smithii*. In 2016, photos taken captured the same trees and showed the dramatic change in tree health with considerable dieback on *S*. *corynanthum* and *D*. *humile* and an increase in foliage transparency on an *A*. *smithii* tree. When examining other *A*. *smithii* trees at the site, it was found that there was considerable variability in susceptibility to *A*. *psidii* within the species.

**Fig 7:**
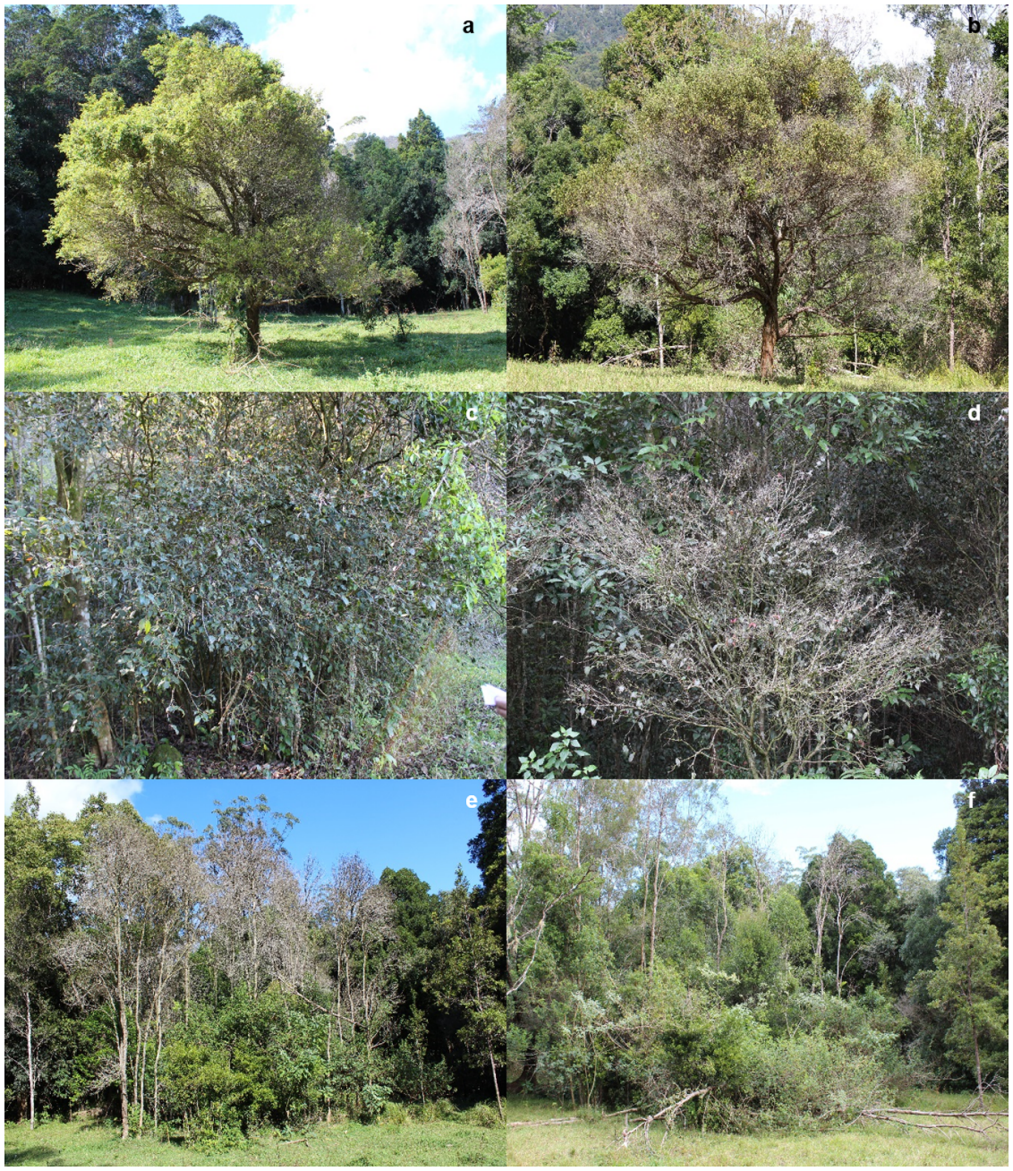
Decline in tree health over time as a result of repeated *Austropuccinia psidii* infection on *Rhodamnia rubescens* (a 2014; b 2016), *Rhodamnia maideniana* (c 2014, d 2016) and *Rhodomyrtus psidioides* (e 2014, f 2016).

In 2014, as part of the studies published by Carnegie *et al*. (2016), surveys included assessments of *Rhodamnia maideniana* but data was not published. In 2014, the average transparency score, an indicator of crown loss and dieback, for twenty *R*. *maideniana* trees at the Ryans Road site was 68.75^±3.55^, increasing to 91.34^±1.51^ in 2016 (Table 3). In 2014 there was no tree mortality as a result of *A*. *psidii* infection in comparison to 29.8% of trees assessed as being dead in 2016 (Fig 7). When comparing *P*. *psidii* infection and impact levels at selected areas across the native range in north east NSW–south east Queensland in 2014 (Table 3), disease incidence levels were similar apart from Hogans Scrub, where disease incidence was significantly lower (F_5,76_ = 12.65; P<0.0001) than other sites assessed. Transparency levels were highest at Tallebudgera Valley (F_5,76_ = 4.84; P<0.0001).

In 2014 96.7% of *Rhodomyrtus psidioides* trees assessed at Ryans Road were dead, increasing to 100% in 2016. No evidence of root sucker regeneration or seedling germination was found at spots where *R*. *psidioides* trees had been killed by *A*. *psidii*. *Rhodomyrtus psidioides* at Ryans Road has been replaced by other species including the noxious weeds lantana (*Lantana camara*) and wild tobacco (*Solanum mauritianum*) (Fig 7).

In 2014 25% of *Rhodamnia rubescens* trees assessed at Ryans Road were found to be dead, with only a 5% increase when assessed again in 2016. However, decline in tree health was observed with foliage loss occurring primarily from the lower branches (Fig 7). Seedling germination/regeneration was not observed under or near any of the trees within the site, nor was there evidence of flower or fruit production on the trees assessed.

## Discussion

*Archirhodomyrtus beckleri*, *Decaspermum humile*, *Gossia hillii* and *Rhodamnia maideniana* were the most common mid- and under-story species of Myrtaceae. All these taxa were significantly impacted upon by *A*. *psidii* with branch death and dieback recorded on all trees, with significant increases in mortality for *A*. *beckleri*, *D*. *humile* and *G*. *hillii*. It is likely that in the very near future these species will become extinct from this location (extirpation) with no evidence of resistance identified in established populations during this study, nor evidence of resistant seedling recruitment. Tree deaths in the twelve months since the plots were first established have more than doubled for *A*. *beckleri*, *D*. *humile* and *G*. *hillii*, suggesting a dramatic decline. This will be a rapid change in the plant community structure, given that the disease was only detected in the region five years prior to our assessment [17,18]. The presence of plant species favoured by disturbance (e.g. *Neolitsea dealbata* http://keys.trin.org.au/key) is further evidence of the impact *A*. *psidii* has caused to this native ecosystem. To fully understand the changes in these ecosystems there is a need for continued monitoring at this site and establishment of long term monitoring plots in other regions.

This is the first report on the impact of *A*. *psidii* at a plant community level in Australia and within a wet sclerophyll ecosystem with a rainforest under-story dominated by Myrtaceae. In Queensland, wet sclerophyll forests are mostly found in the south east but also occur as narrow ecotones bordering the western edge of rainforests in the wet tropics [22]. These ecosystems are unique to Australia [23] and the understory may be comprised of rainforest plants or be grassy with a sparse shrub layer or a combination of both. Species composition may vary depending on climate, topography, soil type and previous land management practices. In the absence of fire or other disturbances, many wet sclerophyll sites will transition to rainforest with a dense understory that reduces light levels, preventing further recruitment of eucalypt species [24]. In the absence of *A*. *psidii*, it would appear that this process was well underway at our study site.

While the overstorey Myrtaceae, *E*. *grandis* and *L*. *confertus*, showed no evidence of *A*. *psidii* impact, all mid- and under-story Myrtaceae showed impacts ranging from foliage loss to tree death. Decline and death of trees and shrubs (*R*. *rubescens*, *R*. *psidioides*) as a direct result of repeated infection by *A*. *psidii* has been demonstrated previously [17,18], but here we report decline and death of a broader range of Myrtaceae (viz. *A*. *beckleri*, *D*. *humile*, *G*. *hillii*). Only *Acmena smithii* showed significant variability in susceptibility to *A*. *psidii* at our study site, with levels of impact significantly lower than recorded for other species. *A*. *smithii* is now the dominant regenerating species based on seedlings present.

Assessments at the two comparison sites suggest that the level of decline observed in our main study site is representative of the impact *A*. *psidii* is having on a broader scale, at least where species composition and stage of transition of the plant community is similar. However, further surveys are required to determine if this type of impact is restricted to the Tallebudgera Valley or if it extends into other areas of similar climate in the local region or other subtropical ecosystems in Queensland and New South Wales. It is also unknown if the same level of impact can be identified in more established rainforest ecosystems in the region. The impact of *A*. *psidii* on more established rainforest trees within the site would suggest that this is quite likely, but that the rate of decline may be slower.

Time will tell if the disturbance caused by *A*. *psidii* is enough to prevent our study site from transitioning to a rainforest ecosystem. Indications are that *Acmena smithii* will become a dominant species, but non-Myrtaceae species may replace those most affected by *A*. *psidii*. Carnegie *et al*. [18] reported that lantana colonized sites where *A*. *psidii* had killed *R*. *psidioides*. This will still represent a dramatic change in species composition and is likely to reduce species diversity within the site. Invasion by lantana affects regeneration of native vegetation, ultimately affecting species diversity of under-story, mid-story and canopy species [25,26]. The long-term impact of a rapid change in species composition at this site, and others of similar composition, is unknown. Studies in other ecosystems, however, have shown that a reduction in biodiversity increases ecosystem vulnerability to invasive plant species and also enhances the spread of plant fungal diseases [27]. There is also likely to be an additional impact on richness and structure of insect communities [27]. Mammals dependent on these insect populations and those that may have relied on the rich variety of food source provided by the diverse Myrtaceae flowers and fruit will be affected. Conversely, a loss of highly susceptible species may see a reduction in disease pressure through lower *A*. *psidii* inoculum levels resulting in reduced disease incidence and severity on less susceptible species.

Given that species like *A*. *beckleri*, *G*. *hillii* and *D*. *humile* are considered widespread (www.ala.orgcom.au), it is possible that the impact we have seen at the site in Tallebudgera Valley is an indicator of the potential long term impact *A*. *psidii* may have on these species across their natural range, similar to that seen for *R*. *psidioides* and *R*. *rubescens* [18]. However, the influence of different climatic conditions on disease incidence and severity may prevent the level of damage seen at this site from occurring at other locations. *Archirhodomyrtus beckleri* has a disjunct distribution with a population in the south occurring from Williams River in New South Wales to Kin Kin in south east Queensland [28], and the northern population extending from Eungella to Mt Lewis in far north of Queensland. The species grows in rainforests on a variety of sites and can also grow as an edge species or as an understory tree in wet sclerophyll forest dominated by *Eucalyptus grandis* as we have seen at our study sites. The species is favoured by disturbance and is a typical re-growth species. A more extensive assessment across the populations would be required to not only determine impact but also identify any possible resistance. The conservation listing for *A*. *beckleri* is currently ‘Least Concern’ (http://www.ehp.qld.gov.au/wildlife/threatened-species; http://www.ala.org.au).

*Gossia hillii* occurs from north east Queensland extending south to north eastern New South Wales and grows as an understory tree in well-developed upland and mountain rain forests. While previously identified as being highly-to-extremely susceptible to *A*. *psidii* [17], our study is the first report on the impact myrtle rust is having on this species in native ecosystems, although anecdotal reports of decline have been made from seed collectors in regions around the Sunshine Coast (http://www.seedpartnership.org.au/partners/qld-bbg/southernpenda). The rate of decline and lack of evidence of regeneration at our study site would suggest that *G*. *hillii* will become less common in the short-term. Like *A*. *beckleri*, the current conservation status of *G*. *hillii* is still listed as Least Concern (http://www.ehp.qld.gov.au/wildlife/threatened-species; http://www.ala.org.au).

*Decaspermum humile* has a wide distribution with a disjunct northern and southern population, occurring from Cape York Peninsula to Townsville in the north, and Bundaberg in Queensland to Wyong in New South Wales in the south (http://www.ala.org.au). *Decaspermum humile* grows in well-developed rain forests on a variety of sites ranging in altitude from near sea level to 1000 m. There is conjecture that the northern population may be considered a separate species (pers. comm. Gordon Guymer). Pegg *et al*. [17] considered the northern form to be different, rating it as being Relatively Tolerant to *A*. *psidii* in comparison to the southern form, which rated as Extremely Susceptible.

The impact of *A*. *psidii* on another common species *S*. *corynanthum*, is significant and has not been reported previously, with ratings of ex-situ plantings indicating the species was Relatively Tolerant to *A*. *psidii*. *Syzygium corynanthum* occurs in north east and south east Queensland and north east New South Wales (http://www.ala.org.au). While the rate of decline of trees assessed at our study site is variable, infection of new growth flush was found on all trees. The severity of impact that *A*. *psidii* will have on this species may not be known for some years.

*Syzygium hodgkinsoniae*, a rare subtropical rainforest tree, grows on alluvial soils by streams in north east New South Wales and south east Queensland, Australia. It is currently listed as Vulnerable (http://www.ala.org.au). The impact of *A*. *psidii* was more severe on juvenile trees than mature trees. Infection occurred on all new growing tips and severe dieback and thinning of the tree canopy resulted from repeated infection. While dieback was present on the mature trees assessed, it was generally restricted to the branch tips. The results of our study, suggest that the impact of *A*. *psidii* is likely to push *S*. *hodgkinsoniae* closer to extinction but more extensive assessments across its range are required.

*Rhodamnia maideniana* has a restricted range in northern New South Wales and south east Queensland, existing within a region considered ideal for *A*. *psidii* [29,30]. It is considered a rare or threatened Australian plant (ROTAP), although its official listing is Least Concern (http://plantnet.rbgsyd.nsw.gov.au/cgi-bin/NSWfl.pl?page=nswfl&lvl=sp&name=Rhodamnia~maideniana). Severe dieback was common on all trees assessed within our trial site and additional sites in northern NSW. Rates of tree death have increased rapidly in the Based on evidence collected from our study, and previous observations [17], we would suggest that the species is likely to be in rapid decline and conservation strategies be implemented while living specimens can still be found.

The rate of decline and change that has occurred at our study site is of great concern. More extensive assessments, both at a species and plant community level, are required to determine if the impact we have seen is representative of these species and plant communities more generally. Monitoring these communities over a greater time period will also provide a better understanding of the long term impact of *A*. *psidii* and consequences of changes at the plant community and ecosystem level. Our studies would suggest that at least four species (*A*. *beckleri*, *D*. *humile*, *G*. *hillii*, *R*. *maideniana*) of Myrtaceae are in severe decline with the likelihood that *S*. *hodgkinsoniae* will be pushed closer to extinction. This adds to previous work identifying *R*. *rubescens* and *R*. *psidioides* in severe decline because of *A*. *psidii* [18] (NSW Scientific Committee). Surveys across other populations and other sites need to be conducted to identify areas at risk and also assess for the potential to select for resistance. This will also provide information for determining any future conservation status for species affected. More information is required from a range of sites to enable more accurate modelling of short and long-term impacts and for determining sites at greatest risk of significant change to plant community structures.

## Supporting information

S1 Table ANOVA table for disease impact across Myrtaceae comparing species within plots and comparison of disease impact levels within Myrtaceae species. (DOCX)

S1 File *Austropuccinia psidii* impact field data. (XLSX)
