# Supplementary material for: Impact of Austropuccinia psidii (myrtle rust) on Myrtaceae-rich wet sclerophyll forests in south east Queensland
Source: PLoS One. 2017 Nov 21;12(11):e0188058. doi: 10.1371/journal.pone.0188058 (PMC5697815; doi:10.1371/journal.pone.0188058)
Supplement: S1 Table — (DOCX) [file pone.0188058.s001.docx]

**ANOVA table for disease impact across Myrtaceae comparing species within plots**

| **Canopy Position** | **Assessment category** | **DF** | **Residual** | **F-value** | **P-value** |
| --- | --- | --- | --- | --- | --- |
| **Regeneration** | Branch death | 4 | 94 | 5.64 | 0.0004 |
|  | Branch dieback | 4 | 94 | 120.44 | <0.0001 |
| **Under-story** | Branch death | 4 | 92 | 9.36 | <0.0001 |
|  | Branch dieback | 4 | 92 | 12.75 | <0.0001 |
|  | Transparency | 4 | 84 | 20.87 | <0.0001 |
| **Mid-story** | Branch death | 3 | 82 | 23.21 | <0.0001 |
|  | Branch dieback | 3 | 82 | 16.23 | <0.0001 |
|  | Transparency | 3 | 82 | 14.4 | <0.0001 |

**ANOVA table for comparison of disease impact levels within Myrtaceae species**

| **Species** | **Assessment category** | **DF** | **Residual** | **F-value** | **P-value** |
| --- | --- | --- | --- | --- | --- |
| ***Acmena smithii*** | Branch death | 2 | 87 | 6.15 | 0.0032 |
|  | Branch dieback | 2 | 87 | 3.41 | 0.037 |
|  | Transparency | 1 | 19 | 1.83 | 0.19 |
| ***Archirhodomyrtus beckleri*** | Branch death | 2 | 81 | 7.05 | 0.0015 |
|  | Branch dieback | 2 | 81 | 4.75 | 0.011 |
|  | Transparency | 1 | 66 | 5.65 | 0.02 |
| ***Decaspermum humile*** | Branch death | 2 | 30 | 20.95 | <0.0001 |
|  | Branch dieback | 2 | 30 | 3.0 | 0.065 |
|  | Transparency | 1 | 25 | 3.64 | 0.07 |
| ***Gossia hillii*** | Branch death | 2 | 41 | 2.57 | 0.089 |
|  | Branch dieback | 2 | 41 | 2.57 | 0.092 |
|  | Transparency | 1 | 36 | 1.85 | 0.18 |
| ***Rhodamnia maideniana*** | Branch death | 1 | 29 | 1.18 | 0.29 |
|  | Branch dieback | 1 | 29 | 2.46 | 0.13 |
|  |  |  |  |  |  |
